# Peptide-Based Supramolecular Hydrogels as Drug Delivery Agents: Recent Advances

**DOI:** 10.3390/gels8110706

**Published:** 2022-11-01

**Authors:** Carlos B. P. Oliveira, Valéria Gomes, Paula M. T. Ferreira, José A. Martins, Peter J. Jervis

**Affiliations:** Centre of Chemistry, University of Minho, Campus de Gualtar, 4710-057 Braga, Portugal

**Keywords:** hydrogel, peptide, drug delivery, controlled release, stimuli-responsive, cancer therapy, supramolecular, smart materials, biomaterials

## Abstract

Supramolecular peptide hydrogels have many important applications in biomedicine, including drug delivery applications for the sustained release of therapeutic molecules. Targeted and selective drug administration is often preferential to systemic drug delivery, as it can allow reduced doses and can avoid the toxicity and side-effects caused by off-target binding. New discoveries are continually being reported in this rapidly developing field. In this review, we report the latest developments in supramolecular peptide-based hydrogels for drug delivery, focusing primarily on discoveries that have been reported in the last four years (2018–present). We address clinical points, such as peptide self-assembly and drug release, mechanical properties in drug delivery, peptide functionalization, bioadhesive properties and drug delivery enhancement strategies, drug release profiles, and different hydrogel matrices for anticancer drug loading and release.

## 1. Introduction

In recent years, supramolecular nanostructures—often in the form of hydrogels—have been widely studied owing to their potential applications in many different areas, such as catalysis, nanofabrication, biosensing, tissue engineering and controlled drug delivery [1]. Low-molecular-weight supramolecular hydrogelators are compounds that, upon receiving a stimulus, can form a three-dimensional (3D) network of entangled fibers. These solid-like materials retain considerable amounts of water (often >99 wt.%) while maintaining a distinct three-dimensional (3D) structure [2,3]. Often, the pore-size of such hydrogels allows the diffusion of molecules and the binding of cells (1).

**Figure 1:**
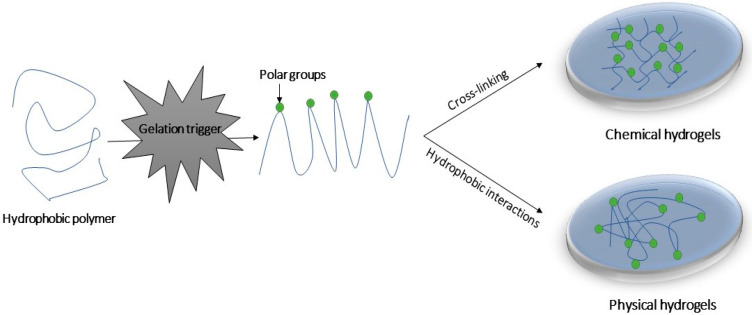
Chemical and physical hydrogels representation.

Hydrogels can be categorized as chemical or physical gels, based on the nature of the cross-linking (Figure 1) [3]. Chemical hydrogels consist of networks connected by covalent interactions. These types of interactions create mechanically resistant and irreversible hydrogels, which undergo significant volume changes during the transition from the solution state to the gel state [3]. The cross-links can be formed in a variety of ways, such as cross-linking by complementary groups, or through the use of high energy radiation, free radical polymerization or enzymes [4,5]. Conversely, physical gels contain networks held together by molecular entanglement and/or secondary molecular interactions (i.e., non-covalent interactions) such as hydrogen bonds, electrostatic interactions and π-π interactions. These interactions can be readily disrupted by changes in the local environment, such as temperature, pH, stress and the presence of specific solutes. Consequently, the formation of physical hydrogels can be reversible, and the transition to the gel state is faster than is observed for the formation of chemical hydrogels [2].

**Figure 2:**
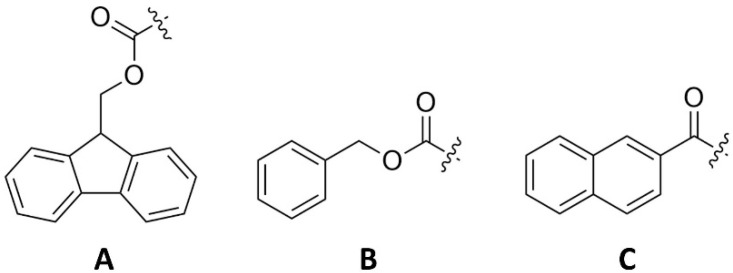
Examples of *N*-terminal aromatic capping groups of peptide hydrogelators: (**A**) Fluorenylmethoxycarbonyl (Fmoc); (**B**) benzyloxycarbonyl (Cbz); (**C**) 2-naphthoyl.

Self-assembly processes derived from biological origins can be potentially exploited in the laboratory setting [6,7]. Peptides and their derivatives are attractive building blocks for the construction of supramolecular hydrogels, as the resulting materials have a high water content and a tunable viscoelasticity, whilst being biocompatible and often injectable [3], thus allowing their application in several fields of biomedicine. The self-assembly process depends on the hydrophobic and hydrophilic characteristics, as well as the hydrogen bond balance of the hydrogelators [7,8]. Peptide-based hydrogelators are usually protected at the *N*-terminus with large aromatic groups, such as fluorenylmethoxycarbonyl (Fmoc), benzyloxycarbonyl (Cbz), naphthoyl or pyrenyl groups (Figure 2) [7].

The unique physical properties of these hydrogels have attracted particular interest for use in drug delivery applications. Conventional drug administration usually requires high doses or repeated administration to stimulate a therapeutic effect, which can result in low overall efficacy and patient compliance, possibly causing severe side effects and/or toxicity [9,10,11]. The most common approach for delivering pharmaceuticals is by oral administration, which is often limited by poor targeting and short circulation times (<12 h) [12]. To address these issues, the development of hydrogels as drug delivery systems is gaining attention, as they can offer spatial and temporal control of drug availability to cells and tissues, thus leveraging the beneficial outcomes of therapeutics by enhancing their efficacy and by reducing their toxicity through a reduction in the required dosage. The highly porous structure of these hydrogels permits the loading of drugs into the gel matrix. Subsequent drug release then occurs at a rate dependent on the diffusion coefficient of the small molecule or macromolecule through the gel network. This porous structure can be tuned by controlling the density of the cross-links in the gel matrix and the affinity of the hydrogels for the aqueous environment in which they are swollen.

The use of hydrogels for drug delivery confers many advantages. From a pharmacokinetic perspective, a depot formulation can be created, from which drugs slowly elute. A high local concentration of drug in the surrounding tissues can then be maintained over an extended period of time [10]. In addition, hydrogels are very biocompatible, owing to their high water content and their physiochemical similarity—both compositionally and mechanically—to the native extracellular matrix (ECM). Hydrogels can be designed to be biodegraded via enzymatic and hydrolytic pathways or by pH, temperature, or electrical fields. Furthermore, these hydrogels are readily deformed, which means that they can adapt to the shape of the surface to which they are applied. These bio-adhesive properties of hydrogels can be advantageous when the gels are applied to irregularly shaped surfaces. For example, the intestinal epithelium and mucosa are biological barriers that are usually wet, dynamic and slippery, which presents a challenge for good adhesion. It is reported in the literature that hydrogels containing 3,4-dihydroxy-L-phenylalanine (L-DOPA) can adhere to epididymal fat pads and external liver surfaces for up to a year, thus promoting bio-adhesion [13,14,15].

Despite these advantages of hydrogels for drug delivery applications, there are still some limitations to consider. The low tensile strength of many hydrogels can limit their use in load-bearing applications and can result in the premature dissolution, or flow away, of the hydrogel from the site of targeted local delivery. In addition, both the overall quantity of drug that can be loaded into the hydrogel, and the degree of homogeneity of the drug loading, can be too low. This is particularly relevant when hydrophobic drugs are to be administered. Large pore sizes combined with the high water content of some hydrogels can result in a rate of drug release that is too fast, perhaps occurring over a few hours. These limitations restrict the use of hydrogel-based drug delivery therapies in clinics.

Several comprehensive reviews have been published in this area [16,17,18,19], while new discoveries are continually being reported in this rapidly developing field. Thus, in this review, we focus primarily on the developments of supramolecular peptide-based hydrogels for drug delivery that have been reported in the previous four years (2018–present) where possible—although older papers will be referred to for the purpose of introducing a topic or providing a historical context. Herein, we address clinical points, such as peptide self-assembly and drug release, mechanical properties in drug delivery, peptide functionalization, bioadhesive properties and drug delivery enhancement strategies, drug release profiles, and different hydrogel matrices for anticancer drug loading and release.

## 2. ‘Smart’ Peptide Hydrogels: Triggers for Self-Assembly and Drug Release

Many peptide hydrogels can be categorized as ‘smart materials’—materials whose properties can be manipulated through their response to an external trigger [20]. An increasing number of studies are examining the effect of the amino acid sequence, chemical modification and mechanical tuning on the behaviour of drug-loaded hydrogels. The study of thermodynamics in the self-assembly process of hydrogel-forming peptides is of great importance [21], as the drug delivery process depends not only on the diffusion of drugs in the hydrogel mesh, but also on nanofiber dissociation [22]. There is agreement regarding the general mechanism of the association of the peptide fibres. It includes monomer conformational changes and hierarchical self-assembly—nuclei genesis followed by their growth into fibrils—culminating in the lateral association of one-dimensional structures [22,23,24]. However, it should be noted that the mechanistic description for the assembly of peptide–drug conjugates is more complex than that of pure peptides, since the typical properties of drugs are quite different from those of amino acid residues. Thus, additional molecular interactions and morphologies need to be accounted for [25,26,27].

**Figure 3:**
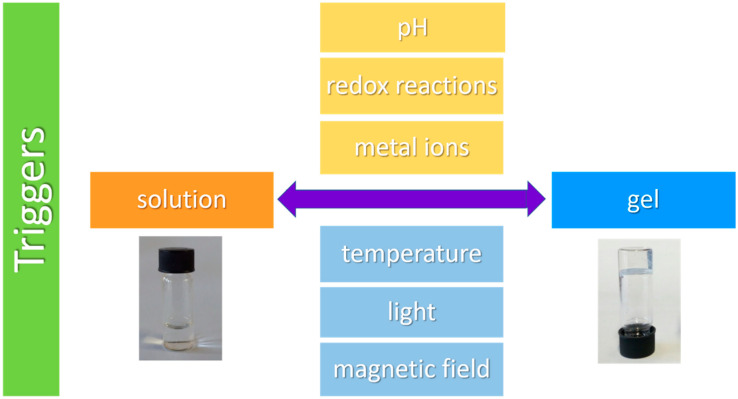
Common triggers for peptide self-assembly and disassembly. Gelation triggers shown in yellow involve a chemical input, whereas gelation triggers shown in light blue do not require a chemical input (i.e., can be manipulated remotely).

Most of these materials load and release drugs in the presence of certain stimuli [27,28] (‘smart’ peptide hydrogels—vide supra) and therefore the following sections are dedicated to explaining the main chemical and physical self-assembly mechanisms (Figure 3) by which peptides can form vehicles for drug delivery applications.

### 2.1. pH-Responsive Gels

In the context of controlled drug delivery, peptides that respond to a change in pH are of particular interest, considering the demand for effective cancer therapies [28,29]. Peptide hydrogels that can trap drugs at a higher pH than the one at which they are released are suitable for carrying chemotherapeutics, since these systems can have selectivity for cancer cells, which have an acidic pH [29,30,31]. The ability of a hydrogel to respond to the local environment can allow selectivity in terms of the temporal and spatial control of drug release, allowing reduced side-effects owing to a sustained local release. They are often easy to prepare, since their synthesis generally only requires acid-sensitive linkers, ionizable groups or specific peptide sequences [32,33]. As the fibres are made up of amphiphilic molecules, the charge on the peptides is highly influenced by the pH of the medium, which can lead to the association or disassociation of the monomers. These hydrogels, with their pKa values close to the pH of the tumour microenvironment, have their pH sensitive moieties protonated in acidic environments, altering the balance between hydrophobic and hydrophilic interactions, and hence the fibrous network of the hydrogel can be broken, promoting the release of the loaded therapeutic agents [32,33,34]. Taking advantage of this, Raza et al. developed the FER-8 peptide (FEFERFK), composed of basic, acidic and hydrophobic amino acid residues [32]. A stable hydrogel was obtained at pH 7.4, since there was no electrostatic repulsion in the absence of charge. Interestingly, it was found that the lower the pH, the higher the rate of decomposition of the gel due to the repulsion between glutamic acid residues. It was possible to incorporate the well-known anticancer drug, paclitaxel (PTX). Studies showed that the FER-8-based hydrogel allowed drug accumulation and a prolonged retention in the desired location, resulting in an overall improved antitumour activity of PTX. In another study, Ghosh prepared a hexapeptide (PEP-1) containing residues of asparagine, lysine, aspartate, leucine, phenylalanine and alanine [31]. Despite failing to provide a gel at acidic and basic pH, the gel could be assembled at physiological pH and showed a large content of β-sheets. At pH = 5.5, the aspartate residues were protonated and therefore repelled by the cationic lysine, while at lower pH values, the electrostatic repulsion between negatively charged aspartate residues discouraged self-assembly [35,36]. To simulate the entrapment and release of a drug, calcein was used. This molecule was incorporated into the gel and released more quickly at acidic pH by virtue of the dissociation of the peptide fibres.

Recently, Kaur et al. introduced π-π interactions into the shortest ionic complementary peptide sequence known (FEFK, a non-gelator) by capping the *N*-terminus with a 2-naphthoxyacetyl group. This modification was sufficient to induce its gelation [37]. This amphiphilic molecule proved to be a hydrogelator over the entire pH range of 2.0–12.0; however, due to the presence of ionic residues, the hydrogel self-assembly behaviour and mechanical properties proved to be highly influenced by pH. At neutral pH, the formed hydrogel showed excellent mechanical properties owing to the saline bridge formed between glutamic acid and lysine (oppositely charged). However, towards the extremes of the pH range, the gels proved weaker, owing to the electrostatic repulsion between the side chains of the monomers [37]. In the future, this work could be applied to pH-dependent controlled drug delivery, or to the construction of several nanostructures obtained from the same compound with mouldable properties.

In cancer therapy it is often desirable to administer two drugs simultaneously, and the two cancer drugs should reach the tumor cells at the same time. Liu et al. recently reported their injectable, pH-responsive peptide hydrogel (VKVKVOVK–V^D^PPT–KVEVKVKV–NH_2_, named ‘OE’), consisting of alternate polar and non-polar amino acid residues. The peptide OE allowed the concurrent delivery of gemcitabine (GEM) and paclitaxel (PTX). At pH = 5.8, this co-delivery system released 97% of PTX after 7 days, compared with only 39% at pH = 7.4% over the same time period. For the release of GEM, almost 100% of the cargo was released after 3 days at both pH = 5.8 and pH = 7.4 [38].

Chen et al. reported uncapped pentapeptides which were able to form hydrogels under pH control. Chen was able to encapsulate cumarin with high photo-thermal protection. The self-assembly process depends on the ionization state of the charged groups present on the amino acid residues and the interactions between amine and carboxylic acid functional groups. Gelation is optimal for the pentapeptide EIWLK, where both ends of the peptide contain additional charged groups. It would be interesting to study the release kinetics for these peptide systems [39].

In a different approach, Yamamoto studied intracellular self-assembly induced by the low pH of the cell microenvironment. The self-assembly of peptide C_16_–VVAEEEE is highly responsive to small changes in pH. Using HEK293 cells, the authors demonstrated intracellular self-assembly. Furthermore, the authors extended the study to show a high anti-tumor activity in vivo [40].

### 2.2. Temperature-Responsive Gels

The use of thermo-responsive supramolecular peptide hydrogels is not widely explored, perhaps owing to the denaturing effect of temperature on these materials [40]. The thermal stability of β-sheets is controlled by non-covalent interactions, including hydrogen bonds between adjacent amino acids in the fibrils. Temperature-sensitive hydrogels assembled in the body can often reverse the sol–gel state in locations containing a large quantity of water [41,42]. In fact, this type of hydrogel is advantageous when used in an injectable system. In the sol phase, the solution embodies the drug, before converting to the gel phase upon injection into the target tissue as a response to body temperature. Hence, these materials are proving to be promising platforms for controlled drug release [43]. In order to confer thermo-responsive behaviour onto the peptide hydrogelator I_3_K (IIIK), Meiwen Cao functionalized the peptide with poly(*N*-isopropylacrylamide) (PNIPAM) [43]. The composite showed a reversible sol–gel phase transition at a point close to physiological temperature (33 °C), where conformational changes were detected. As a result of the hydrogel’s ability to load an antimicrobial peptide and release it above a lower critical solution temperature, it is believed that this mixture can act as a non-invasive drug delivery nanosystem. In an elegant approach, de Leon-Rodriguez et al. described the synthesis of a hydrogelator consisting of a HβP peptide (PEELKLELKLEP or PEELELELKLEP), the metalloproteinase 2 (MMP2) substrate peptide sequence (IPESLRAG) and the RGD cell adhesion epitope (GRGDSP–NH_2_) [44]. The peptide that contained a HβP portion with two lysine residues peptide showed a higher β-sheet content with increasing temperature, which points to energy-dependent self-assembly. Thus, it is concluded that the sol–gel transition results from conformational changes (the conversion of random coils into β-sheets). In addition to having their state of association governed by temperature, both hydrogels are degraded in a controlled manner by MMP2, rendering them interesting candidates for drug delivery [44].

### 2.3. Redox-Responsive Gels

Although oxidation/reduction reactions have not yet been widely applied in responsive supramolecular peptide hydrogel systems, they are proving a very useful tool for modulating the behaviour of these materials in drug delivery settings. As redox-responsive hydrogels containing disulfide bridges would be effectively degraded in the presence of glutathione (GSH), this type of gel is an encouraging idea to selectively release anticancer drugs, since tumours are marked by highly reductive environments [45,46]. In 2020, the IC1–R–PTX peptide conjugate (CKIKIKIK–IDPPT–KIOIKIKC–NH_2_–paclitaxel) was reported, which, in addition to being pH-responsive, showed sensitivity to reducing agents [47]. The peptide formed a stable gel at neutral pH but remained in the form of solution in a slightly acidic medium (pH 6.4). After seven days, there was a cumulative drug release of 66% at pH 5.8. To study the redox-responsive properties of the IC1–R–PTX-based hydrogel, different media were used; a higher PTX release rate was observed in a medium containing GSH at pH 5.8, compared to media with neutral pH and/or without GSH. In addition to these positive results, Zhu found that the rate of drug release was tuneable through changes to the peptide concentration, i.e., the higher the concentration of hydrogelator, the lower the rate of PTX release rate, thus maintaining a doubly responsive profile [47]. In an alternative system, Wu et al. produced a hydrogelator precursor, HCPT–SA–FFE–ss–EE, in which “HCPT” corresponds to the anticancer 10-hydroxycamptothecin and “ss” represents a disulfide bridge between the glutamic acid residues [48]. The reduction of the latter by GSH allows the self-assembly of the monomers, since it decreases peptide hydrophilicity, thus producing a hydrogel with thixotropic behaviour. There was no burst release of HCPT, and the sustained drug delivery was a result of two processes: pharmaceutical diffusion in the gel matrix and hydrolysis of the ester link between HCPT and the peptide chain [48]. The reader should note that there are several viable pathways for achieving the same goal: Zhu’s work uses the reductive ability of GSH to trigger the drug release, while Wu’s work uses the same process to induce self-assembly of the drug–peptide conjugate in order to avoid HCPT burst release. Both approaches are attractive for cancer treatment using injectable materials.

### 2.4. Metal Ion-Responsive Gels

Hydrogel design in biomedicine is aimed towards translation into clinical practice, and the location of specific ions in certain tissues can be used to trigger the formation of peptide supramolecular hydrogels [49]. The recently reported ion-π interactions are influenced by ion charge and the type of aromatic system used. Thus, metal ions are crucial elements in the association of some low-molecular-weight peptides, changing their conformational structures and, therefore, their biochemical effects [50,51]. In order to treat microbial infections, D’Souza reports the synthesis of the L9 peptide (Ac–(3′-PyA)LRLRLRL(3′-PyA)–CONH_2_), which binds Ag(I) ions and produces hydrogels 15 times stiffer (with a higher content of β-sheets) than those formed in the absence of silver [52]. These platforms were found to release Ag(I) in sufficient quantities to display antimicrobial activity. Arginine residues improve peptide bactericidal activity, while the pyridyl alanine versions allow coordination with silver ions, as a result of their low pK_a_ value. The amount of Ag (I) used should be minimized to avoid toxicity in mammalian cells. Following charge screening, it was found that sodium nitrate could be successfully used to repair the lost stiffness. It followed that this pH-independent nanosystem showed great potential in wound healing [52,53]. In an impressive work, Tao’s team designed the peptide E_3_F_3_ (EEEFFF), whose gelation properties were enhanced in the presence of zinc ions [54]. Increasing zinc concentration boosted E_3_F_3_ self-assembly as a result of a greater probability of chelation, leading to stronger π–π interactions. These forces acted in synergy with the exposed carbonyl groups, which were available to coordinate zinc. The drug delivery ability of this system was studied using an anticancer compound, and a slow drug release was observed, in addition to an increase in its antitumor effect. As the prostate is especially rich in these ions and the E_3_F_3_ peptide gelation occurs in a few minutes, this system allows the encapsulation of drugs in situ [51,54].

### 2.5. Light-Responsive Gels

Another method for inducing peptide self-assembly and drug release is through the use of light. There are two types of light-responsive hydrogels: photodegradable hydrogels, which contain photo-labile groups, and thermo-sensitive hydrogels, which contain NIR-absorbing portions [55]. The former allows drug release through the dissociation of fibres, and the latter can release drugs through volume changes caused by dimerization and/or polymerization [56]. There are some limitations to the use of UV light, such as a limited depth of penetration into tissues, and the fact that prolonged exposure to UV rays can cause cell damage. However, this trigger is still widely studied because of the possibility of remote temporal and spatial control of drug delivery [55,57]. As an example of a photodegradable hydrogelator, the peptide FmocFFpS^C^(oNB)–PEG was constructed, in which “pS^C^(oNB)” represents a di-*o*-nitrobenzyl-protected phosphonated serine residue and “PEG” corresponds to a carboxylic acid attached to a polyethylene glycol chain [58]. This amphipathic molecule was able to form to a hydrogel at neutral pH in the presence of calcium ions, which coordinated the negatively charged carboxylic acid. A total of 90% of the photoactive *o*-nitrobenzyl protective groups were degraded in the presence of UV light and the negative charge of the phosphonates was exposed within 2 h, leading to fiber degradation. When doxorubicin (DOX) was added to the hydrogel precursor solution, this drug could be encapsulated following the addition of Ca^2+^ ions. More importantly, during UV irradiation, the fibers dissociated and provided rapid drug release, with the drug proving stable to UV exposure [58].

Guilbaud-Chéreau et al. developed Boc-protected dipeptide hydrogels based on Boc-α-diphenylalanine and its β- and γ-homologues. The incorporation of carbon nanotubes (CNTs) or graphine oxide into the gel structures rendered them photo-responive―-treatment with NIR light triggered the degradation of the hydrogel. This process was applied to the on-demand release of ascorbic acid. Interestingly, the same structures containing tyrosine instead of phenylalanine failed to produce hydrogels, indicating a detrimental influence of the phenol OH group on the self-assembly process [59].

**Figure 4:**
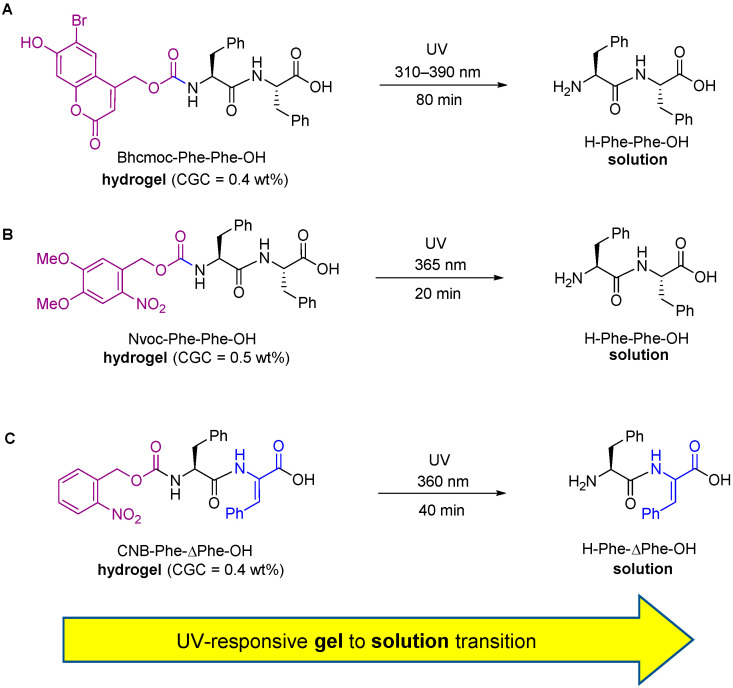
Sol-to-gel transitions of light-responsive hydrogels capped with photo-labile groups: (**A**) dipeptide capped with Bhcmoc; (**B**) dipeptide capped with Nvov; (**C**) dipeptide capped with CNB.

As mentioned previously, short peptides are usually capped on the *N*-terminus with an aromatic group. When designing UV-responsive hydrogel systems involving short *N*-capped dipeptides, the aromatic capping group required for self-assembly can often be switched for a photo-cleavable version of the capping group. A short exposure of the hydrogel to UV light is able to cleave the capping group, and a peptide is released that is more aqueous-soluble than the starting material. This photolysis reaction is accompanied by a loss of the π-π stacking interactions essential for self-assembly. The nanostructures holding the hydrogel network together are therefore disrupted, resulting in a gel-to-sol, or gel-to-solution, transition. This concept was originally explored by Hamachi et al. [60], and then later applied to drug delivery applications by Shabat and Adler-Abramovich [56]. Hamachi discovered that diphenylalanine *N*-capped with a photo-cleavable coumarin derivative (Bhcmoc–Phe–Phe–OH) could form a hydrogel. The critical gelation concentration (CGC) of the gel was determined to be 0.4 wt.%. Exposure to UV light (310–390 nm) resulted in a gel-to-sol transition within 80 min (Figure 4A) [60]. Hamachi later modified this compound to create a two-photon responsive hydrogel, which was responsive to near-infrared (NIR) light (740 nm) [61]. Adler-Abramovich investigated a different photo-responsive system, involving a nitroveratryloxycarbonate-capped dipeptide, Nvoc–Phe–Phe–OH (Figure 4B) [56]. This compound formed a hydrogel with a CGC of 0.5 wt.%. A gel-to-sol transition of this hydrogel occurred in 20 min upon irradiation with UV light (365 nm). Importantly, this gel-to-sol transition could be used to accelerate the release of an encapsulated insulin–fluoroscein isothiocyanate conjugate (insulin-FITC) from the hydrogel network, which was released very slowly by diffusion alone. Our own research group used a similar principle to develop a model photo-caged dehydrodipeptide [62]. There are several dehydrodipeptides with biological activity known in the literature, and we were able to show that CNB–Phe–ΔPhe–OH can either be used to either provide the self-delivery of a dehydrodipeptide on-demand, or be used to deliver an antibiotic (ciprofloxacin) (Figure 4C). It follows that a short burst of UV light (360 nm) can be used to provide a partial disruption of the hydrogel and an acceleration of drug release, or a longer treatment with UV light (360 nm) can be used to provide to give a complete release of drug cargo [62].

### 2.6. Other Stimuli

Up to this point we have been addressing the most commonly reported triggers for gel formation and gel degradation. However, there are many more physical and chemical factors which can induce changes to hydrogel properties, both in peptide self-assembly and in controlled drug delivery [63]. An external magnetic field can also act as a trigger for these changes. This application requires the introduction of a different class of materials, the most important being magnetic nanoparticles. Carvalho et al. developed a dehydrodipeptide-based hydrogel with incorporated superparamagnetic iron oxide nanoparticles (SPIONs). The hydrogelators studied contained either a tyrosine or an aspartic acid residue attached to a 1,2-didehydro-phenylalanine residue. The effect of the SPIONs on the self-assembly process, as well as on the mechanical and magnetic properties of the hydrogels, was studied. It was found that the magnetic hydrogels containing SPIONs provided *T*_2_-MRI contrast enhancement in a concentration-dependent manner. Furthermore, magnetic excitation of the SPIONs generated a significant amount of heat, providing a remote trigger for release of drug cargos [64]. In addition, it is estimated that there are several approaches that are yet to be discovered and combined within the known peptide hydrogels themselves. For example, without using new materials, Gayen et al. found that the NDIP peptide (histidine-appended naphthalenediimide) can form gels following the addition of tartaric acid [65]. Intriguingly, it was observed that several hydrogel properties (thermal stability, fluorescence properties and stiffness) were significantly altered upon aging, without the addition of any other component [65].

The action of enzymes within biological systems can also be exploited. In an approach taking advantage of metalloproteinase 7 (MMP7) overexpression in cancer cells, Cao used this enzyme to promote the gel-to-sol transition of Nap–FFGPLGLARKRK peptide [66]. The initial hydrogel was capable of encapsulating large amounts of DOX, which, when exposed to enzymatic action, underwent morphological changes. As the GPLGLA motif is the substrate for MMP7, this peptide cleavage occurred selectively at cancer cells, allowing targeted drug delivery [66]. Another example of an enzyme-mediated release system was reported by Kulkarni, where covalently attached bioactive molecules could be released upon the action of endogenous esterases. More specifically, tripeptides covalently attached to lactose, as well as tripeptides covalently attached to peptidic mimics of bioactive brain-derived neurotrophic factor (BDNF), were prepared. As the conjugation was through ester bonds, the action of serum esterases provided a sustained release of the conjugated molecules. More importantly, the released BDNF-mimics provided increased neuronal survival and improved the normal neuronal function of peripheral neurons [67].

**Figure 5:**
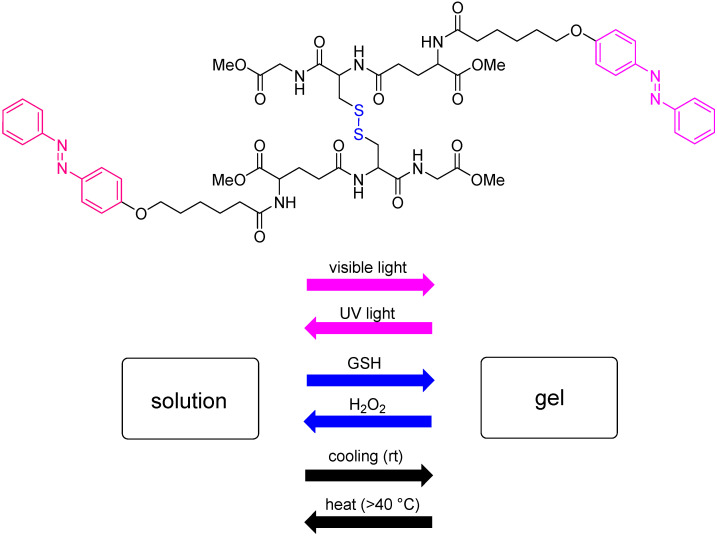
Multi-stimuli-responsive peptide hydrogel reported by Liu et al. (Adapted from [68]).

The future of this area perhaps lies In multi-stimuli responsive systems, where a range of options is available for controlling self-assembly processes, combining the various triggers described in this section. Liu et al. recently reported a thermo-, redox- and light-responsive supramolecular hydrogel that is constructed from an oxidized glutathione derivative. The peptide contains a disulfide bridge and azobenzene moieties, which can be manipulated by GSH/H_2_O_2_ and UV light, respectively (Figure 5). This, and other, multi-stimuli-responsive peptides have clear potential in targeted drug delivery [68].

## 3. Hydrogel Mechanical Properties and Stability for Drug Delivery

As previously mentioned, peptides are versatile building blocks for supramolecular assembly, as they can be assembled from the 20 available canonical amino acids, as well as non-coding and non-natural variants. Their self-assembly is dictated by the primary structure of the peptide. Depending on their molecular design, peptides can adopt various secondary structures, such as β-sheet, α-helix, and β-hairpin structures. There are several reviews in the literature covering different classes of self-assembling peptides [69,70]. A good example is the review of Rodriguez et al. that discusses structure–mechanical property correlations of peptide hydrogels forming β-sheets [71]. As discussed by Rodriguez et al., the softness or stiffness of the hydrogel is a very important characteristic in biomedical applications, and by extension, drug delivery. These physical properties can be optimised by changing the peptide concentration, ionic strength, and pH [71]. However, for some applications in which stiffer hydrogels are required, this may not be enough. One approach to overcome this limitation was recently described by Schneider et al., who utilized Frémy’s salt to increase the stiffness of the supramolecular networks [72]. They report that the addition of Frémy’s salt to their novel gelation system resulted in the conversion of the phenol group of the tyrosine residue into a reactive *o*-quinone group, which readily reacts with the amine groups present on the lysine residues of the assembled peptide, and thus covalent cross-links are introduced into the system. These covalent cross-links strengthen the network and consequently the mechanical rigidity of the hydrogel is increased [72].

### 3.1. β-Sheet Forming Peptides

The simplest self-assembling peptide known is di-L-phenylalanine (Phe–Phe). Reported by Grazit et al., the dipeptide was soluble in organic solvents, including 1,1,1,3,3,3,-hexafluoro-2-propanol. Diluting a solution of the peptide with water to a final concentration of 1 mg/mL resulted in a fast assembly into ordered semicrystalline structures, which could be observed visually [73]. To probe the molecular arrangement of the assembled structure, Fourier-transformed infrared spectroscopy (FTIR) was employed. A sharp peak at 1630 cm^−1^ (amide I region) was observed, suggesting a β-sheet-like conformation of a single amide bond [73]. Later, Grazit et al. reported the thermal and chemical stability of the di-L-phenylalanine peptide gel [74]. The results showed a remarkable thermal and chemical stability of the dipeptide nanotubes in both wet and dry conditions, pointing to their potential nanotechnological applications [74]. The self-assembly of the dipeptide modified with an Fmoc group at the *N*-terminus was also studied [75]. The secondary structures of this modified dipeptide were analyzed by FTIR and circular dichroism (CD), with both revealing β-sheet folding [75]. In addition, π–π interactions between the aromatic groups were indicated by a shift in fluorescence emission from 320 nm to 330 nm [75]. These ultrashort peptides are frequently modified at the *N*-terminus end with aromatic groups, which enhance the gelation process due to the effective π-π interactions [76]. Analogues of di-L-phenylalanine are currently being widely investigated in drug delivery, due to the mechanical and morphological advantages. One example is the work reported by Konar et al., in which fluorinated di-L-phenylalanine analogues were used to sustained release of antineoplastic drugs [77]. They reported the synthesis of two hydrogelators, (**I**) Fmoc–(4-F)–Phe–Phe–OH and (**II**) Fmoc–(3-F)–Phe–Phe–OH, and performed rheological tests to evaluate their mechanical integrity. The data showed that hydrogelator **I** (G’(storage modulus) = 1700 Pa) was a stronger gel than hydrogelator **II** (G’ = 500 Pa) for gels of the same concentration, highlighting the importance of the fluorine position in regulating the mechanical properties [77]. Further analysis employing FTIR and powder X-ray diffraction (PXRD) indicated a β-sheet structure, which is believed to assemble due to the effective π-π interactions between the aromatic rings of the phenylalanine and Fmoc moieties [77].

Another class of peptides that are being widely studied are nanofiber-forming peptide amphiphiles (PAs). PAs consist of a hydrophobic tail, a peptide section with a tunable ability to form β-sheets, a charged section to increase solubility and an attached biologically active moeity [78]. These PAs can self-assemble in water as a result of the hydrophobic collapse of the tails of the first section, as well as β-sheet formation in the second section. The hydrogen bonds in the β-sheet structure promote the linear direction of assembly formation, resulting in a fibrillar architecture [78]. Different sequences of PAs have been designed for different biomedical applications, including drug delivery [78,79]. Recently, Matson et al. reported aromatic peptide amphiphile hydrogels for the controlled delivery of hydrogen sulfide (H_2_S) [80]. H_2_S releasing PA hydrogels consist of a small peptide chain (typically between 2–7 amino acids) with an *S*-aroylthiooxime (SATO) attached to the *N*-terminus via a covalent bonded linker segment. SATOs are aromatic H_2_S donors, triggered by the presence of thiols [80]. Matson et al. report that the stiffer hydrogels provide an overall longer sustained release compared to weaker hydrogels, because the more rigid hydrogels have increased access to cysteine (Cys) in the nanofiber core. These PA-based hydrogels allowed tunable H_2_S release while minimising structural modifications. The flexibility of the linker affected the rheological behavior of the PA gels—peptides with flexible linkers formed stiffer gels, while peptides containing relatively rigid linkers formed weaker gels [80].

The peptide EAK16-II [(Ala–Glu–Ala–Glu–Ala–Lys–Ala–Lys)_2_] is well known for forming fibrils with stable β-sheet secondary structures [81]. Calvanese et al. recently reported modifications to EAK16-II through the incorporation of cysteine residues, thus allowing chemical cross-linking through the formation of disulfide bridges. Analogues where cysteine was attached at one end (C–EAK), and at both ends (C–EAK–C), of the sequence were studied before and after oxidation to their disulfide bridged versions. The oxidized version of C–EAK proved to be the most promising peptide in terms of mechanical properties, biocompatibility and the ability to support the growth of osteographs. Molecular dynamics (MD) simulations supported the fact that one, as opposed to two, cysteine residues would provide the optimal properties [82].

The tunability of these biomaterials is key for therapeutics where localized delivery is crucial. For this reason, systems where the self-assembly pathway can be modified have gained increased attention in recent years. One example of this is the recent work reported by Roy et al., where they explored the effect of pH on the self-assembly pathway and the physicochemical properties of the final gel-phase material [37]. In this work, they used a classical non-gelator with an ionic complementary sequence (Phe–Glu–Phe–Lys) conjugated to a naphthoxyacetate (Nap) group at the *N*-terminus. Ionic self-complementary peptides, inspired by zuotin protein (Z-DNA-binding protein in yeast), undergo a structural switch in response to a pH change [83]. As discussed by Roy et al., the incorporation of oppositely charged amino acid residues into the PA resulted in pH-responsive behavior, leading to the formation of hydrogels over a pH range of 2.0–12.0. However, the mechanical and morphological properties of the hydrogels were significantly different. The presence of charge on the gelator at more extreme pH values led to the formation of thinner fibers, whereas thicker fibers were observed near to physiological pH values. This is due to charge neutralization supporting lateral association. This variation in molecular packing reflects the variations in the mechanical strengths of the peptide gels [37], suggesting that the rigidity of the hydrogels can be tuned by changing the pH. Recently, Banerjee et al. reported that aging can improve the thermal stability, mechanical stiffness and fluorescence properties of histidine-conjugated two-component hydrogels containing naphthalenediimide (NDIP) [65]. The self-assembly of NDIP formed an aggregate unable to form a gel in Milli-Q water at pH 6.6. However, the addition of tartaric acid allowed the formation of a transparent gel, where the initial nanotube structure was converted into a cross-linked nanofibrous structure. Interestingly, the gel properties changed upon aging, as it was found that the thermal stability and the stiffness of the gel increased over time [65]. The rheological experiments showed that the storage modulus G’ increased 146-fold (from 32.7 Pa to 4517 Pa) after aging for 40 days at a fixed angular frequency [65]. These results suggest that intermolecular interactions between the molecules of gelator and tartaric acid in the gel phase slowly increase over time, creating a more mechanically stiff and thermally stable gel.

### 3.2. β-Hairpin Forming Peptides

**Figure 6:**
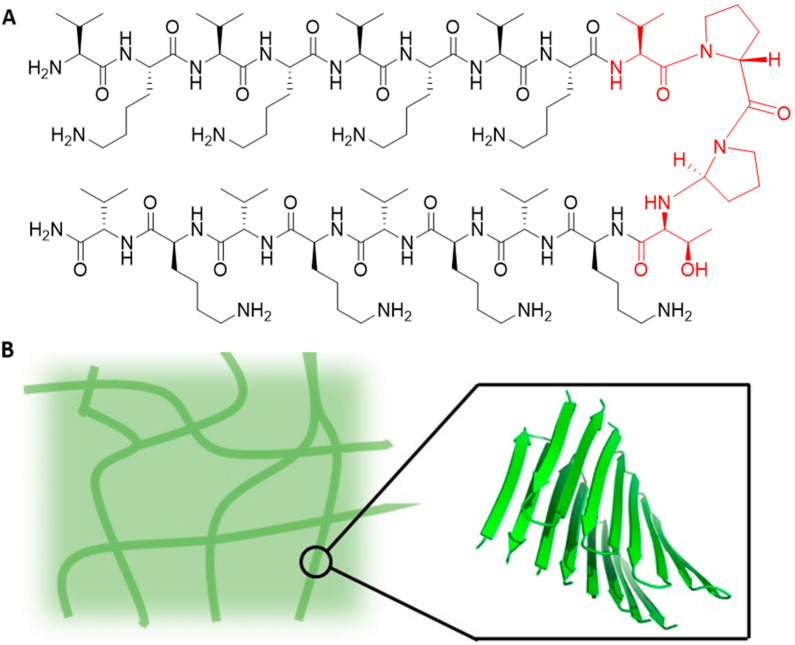
Structure of the MAX1 peptide, composed of alternating valine and lysine residues (**A**) and ribbon representation of the fibrils (**B**).

Schneider and Pochan have reported peptides that can respond to distinct external stimuli and fold into ‘facially amphiphilic’ β-hairpins, and consequently self-assemble into rigid hydrogels [72,73,74,75,76,77,78,79,83,84,85,86,87,88,89]. This class of peptides is sometimes named the MAX1 family, as MAX1 is the parent sequence. MAX1 is a peptide that contains 20 amino acid residues and is comprised of two β-strands containing alternate valine and lysine residues. This amino acid sequence has a high propensity for β-sheet formation. A tetrapeptide (–Val–*D*-Pro–Pro–Thr–) sequence was used to connect the strands in order to introduce a type II’ turn into the structure [88] (Figure 6). Under acidic conditions, the protonated lysine residues prevent peptide folding, and therefore self-assembly does not occur. However, intramolecular folding and subsequent self-assembly can be initiated by either neutralizing the charge by increasing the pH to pH 9, or by masking the positive charges on the lysine residues by the addition of salt at physiological pH [86,88]. The folding process is regulated by the electrostatic interactions of the hydrophilic face of the hairpin, the formation of intramolecular van der Waals contacts between the side chains, and the formation of intramolecular hydrogen bonds between the carbonyl and amide groups of the sidechains of the peptide backbone. The peptide turn regions greatly influence both the structure and function. The types of β-turns are classified according to the main-chain dihedral angles (Φ,Ψ) adopted between the second and third residues. The type of turn influences the β-sheet properties, such as β-sheet twist, stability, folding nucleation rate and the degree of hydrogen bonding [90]. Studies have demonstrated that formation and stability of β-hairpins is influenced by the type of β-turn, and the propensity of the residues at the turn positions to adopt dihedral angles consistent with β-turn structures [91,92].

The mechanical and morphological properties of these peptide-based hydrogels and their stability meet the biological requirements for tissue-engineering applications, such as cell [85] and drug delivery [93]. It should be noted that both MAX1 and MAX8 hydrogels are known for their shear-thinning properties. These gels shear thin when a significant stress is applied to the material and then self-heal when the application of shear stress is removed. This shear-thinning/self-healing behavior constitutes a delivery route for targeted and controlled drug release, as is discussed further in this review.

Recently, Brimble and Cornish et al. designed, characterized and evaluated β-hairpin peptide hydrogels for the delivery of bovine lactoferrin (LF) [94]. Encouraged by the design of MAX1, they studied three related β-hairpin peptide hydrogels. They had already reported the synthesis and characterization of H_2_N–Leu–His–Leu–His–Leu–Lys–Leu–Lys–Val–*D*–Pro–Pro–Thr–Lys–Leu–Lys–Leu–His–Leu–His–Leu–CONH_2_ (H4LMAX) [84]. This peptide is a modified MAX1, where a Val residue is substituted for a Leu residue, and four Lys residues are replaced by His residues, which decreases the electrostatic repulsions of the positively charged residues and allows H4LMAX to form hydrogels at neutral pH without the need for an additional stimulus [94]. H4LMAX was found to be non-toxic, but did not support cell attachment. In a second version, an RGDS (Arg–Gly–Asp–Ser) motif was added to the *C*-terminus of H4LMAX peptide, resulting in H4LMAX–RGDS. The rationale for this design was not only to promote cell attachment but to maximize the presence of alternating hydrophobic/hydrophilic amino acids in the sequence, which is characteristic of β-sheet-forming peptide hydrogelators. This peptide was also found to be non-toxic, and crucially the presence of the RGD sequence permitted osteoblast attachment—although the gel was less stiff than H4LMAX. In the third peptide, His4 was substituted with Asp, and His17 with Arg, to provide the peptide H2LRDMAX. This design placed the Arg and Asp residues facing each other on opposite arms of the peptide in a β-hairpin conformation, in order to mimic an RGD (Arg–Gly–Asp) sequence through space [94,95]. The rheological results showed that H2LRDMAX was a stiffer and faster-forming gel than H4LMAX–RGDS. However, H4LMAX had a negative effect on cell morphology, and therefore only H4LMAX–RGDS was tested in subsequent drug delivery studies. The gel of H4LMAX–RGDS was able to provide a sustained release of lactoferrin (LF), releasing 60% of the cargo over 5 days.

### 3.3. α-Helix Forming Peptides

α-Helix forming peptides have drawn researchers’ attention through the years, as they can form nanostructures similar to those found in the cytoskeleton and the ECM of biological systems [96]. These α-helical peptides with 2–5 helices can aggregate and form nanofibers [97,98]. In addition, peptides constructed from around 30 amino acid residues were also able to form nanostructures through helical coiled-coil structures [99]. Helical peptides with triblock motifs having coiled-coil blocks could also form hydrogels [100]. Tuning the length and structure of the coiled-coil units allows the hydrogel properties to be managed. Therefore, these materials can be used as stimuli-responsive hydrogels for drug delivery applications.

Recently, Chang and Wang et al. reported efficient α-helix cell-penetrating peptides for intracellular cargo delivery [101]. Cell-penetrating peptides (CPPs) are a potential tool for the transport of various biologically relevant molecules, such as functional peptides, proteins, nucleic acids and small-molecule drugs [102]. They investigated the cell-penetrating properties of Tat, which are stapled CPPs designed to exhibit a cationic secondary amphipathic structure [101]. As Chang and Wang et al. discussed, the rigidified α-helix conformation, hydrophobic bridge, and the number and location of cationic groups from helical backbones increased the cellular uptake. Their results also revealed that increased affinity of the rigid stapled Tat peptides to heparan sulfate correlated with increased cellular uptake compared to non-stapled Tat peptides with flexible chains [102]. The stapled Tat peptides showed increased endosomal escape, high proteolytic stability and low cytotoxicity.

## 4. Hydrogel Delivery Routes and Macroscopic Properties for Drug Delivery

The multifunctional properties of hydrogels are vital for their potential applications, which serve to protect, target and locally deliver drug molecules. To better understand this dynamic, this section starts by exploring the delivery routes and the macroscopic properties of hydrogels. Next, we proceed to the mesh scale, which regulates diffusion, and its temporal or stimulus-responsive evolution. Hydrogel delivery systems can be categorized into three groups based on their size: macroscopic hydrogels, microgels and nanogels. Since hydrogels can be applied or formed into almost any shape and/or form, the size of the hydrogel is important. It is the macroscopic design of the hydrogel that determinates the delivery route.

### 4.1. Macroscopic Hydrogels

Macroscopic hydrogels are usually either implanted surgically into the body or placed in contact with the body for transepithelial drug delivery [103]. Their size is typically on the order of millimeters to centimeters. These macroscopic hydrogels can be divided into three categories according to their delivery routes: in situ-gelling gels, macroporous gels and shear-thinning gels.

#### 4.1.1. In Situ-Gelling Hydrogels

These hydrogels can be injected in liquid form and before undergoing a sol–gel transition inside the body. The resulting hydrogels take the shape of the space in which the gel was injected. The sol–gel transition can be achieved through different methods. Slow-gelling systems are one way to achieve this gelation process. In this method, the gelation process is initiated outside of the body. As gelation occurs slowly, the solution can be injected before solidification occurs. This method has been applied to systems using various gelation mechanisms, such as charge interaction [104], stereocomplexation [105] and Michael addition [106]. Another strategy being explored is the development of thermosensitive hydrogels. Injectable thermosensitive hydrogels are promising biomaterials that have a low critical solution temperature (LCST), above which they undergo transition from sol phase to gel phase. Their characteristics allow therapeutic agents to be easily encapsulated by injecting in the solution phase, followed by the formation of the hydrogel in situ at physiological temperature [107].

Recently, Wei and Tang et al. studied the antitumor effects of emodin (EM)-loaded peptide hydrogels in situ [108]. These systems are injectable solutions before administration, and then semi-solid or solid hydrogels are formed in situ at the site of drug administration. This phase transition is stimulated by external conditions, such as light, temperature, or pH. In this particular case, the RADA16–I peptide was used as the gelator. RADA16–I is an ionic complementary self-assembling peptide, and as discussed previously in this review, the gelation is triggered by pH. In this work, the results showed that the RADA16–I–EM hydrogels formed in situ significantly reduced the tumor growth rate and reduced the toxic side effects of EM in normal organs in vivo, compared with the free EM in subcutaneously implanted murine Hepa1-6 liver tumor models. This effect is attributed to the RADA16–I–EM hydrogels, as they effectively deliver EM into the tumor tissue [108]. This work highlights in situ-gelling hydrogels for drug administration and localized sustained drug delivery, while also demonstrating their potential in further biomedical applications.

#### 4.1.2. Shear-Thinning Hydrogels

Some hydrogels can be pre-gelled outside of the body and then injected, through the application of shear stress. These hydrogels flow like a low-viscosity fluid under shear stress during injection, but quickly self-heal after the removal of the shear stress, regaining their initial stiffness. This behavior results from the reversible properties of the physical cross-links. Physical cross-links are reversible due to the dynamic competition between pro-assembly forces—which include hydrophobic interactions, electrostatic interactions and hydrogen bonding—and anti-assembly forces—such as solvation and electrostatic repulsion [109]. As mentioned previously in this review, the MAX1 family has been developed over the years as injectable hydrogels for drug delivery [93,94]. Miller et al. have recently reported a series of novel β-hairpin peptides that bind to Zn^2+^ ions and produce fibrillar structures. They designed nine novel peptides, all based on the MAX1 peptide. Mutations of the Lys and Val residues at different positions along the sequence of MAX1 to His and Cys residues were implemented to create potential binding sites to Zn^2+^ ions. The locations and the number of Cys and His residues in the Zn^2+^ binding site affected the molecular mechanism of self-assembly of the peptide and consequently its structural characteristics [110]. In cases of three or four His residues in the Zn^2+^ binding site, the nanofibers where more rigid and less twisted. Conversely, in cases of two, three or four Cys residues in the Zn^2+^ binding site, the nanofibers were more twisted, flexible and brittle [110].

Although the MAX1 peptide family is very well known for its shear-thinning properties, there are still other supramolecular peptide-based hydrogels that are being investigated for their shear-thinning/self-healing properties. Recently, Bai and Li et al. designed a series of aromatic dipeptides that form shear-thinning hydrogels with self-healing and tunable mechanical properties [108]. They reported the design and synthesis of Fmoc-conjugated Phe–Phe, Tyr–Leu, Leu–Leu and Tyr–Ala. The results showed that a synergic effect of the hydrophobic interactions and hydrogen bonding interactions is a crucial factor affecting the mechanical strength and self-healing properties of hydrogels. By increasing the hydrophobic interactions among molecules, the mechanical stiffness is enhanced, and by increasing the hydrogen bonding interactions, the self-healing process is enhanced [111].

#### 4.1.3. Macroporous Hydrogels

Another approach for preparing injectable hydrogels is to create large hydrogels with interconnected pores that can mechanically collapse and recover reversibly. When the gel is delivered via injection with a needle, water is squeezed out from the pores, and the gel collapses, allowing it to pass through the needle. Once the gel is extruded and the mechanical constraint imposed by the needle walls is removed, the hydrogel recovers its initial shape almost immediately in the body. The behavior of these hydrogels resembles that of foams and they can be reversibly compressed with up to 90% strain without any permanent damage to the gel network [112]. Through the years, many methods have been described for the fabrication of these types of hydrogel, such as gas foaming [113], microemulsion [114], freeze drying [115] and cryogelation [116]. One example is the work reported by Kirsebom et al., in which they described the formation of macroporous self-assembled hydrogels through cryogelation of Fmoc–Phe–Phe–OH [116].

### 4.2. Microgels and Nanogels

Using small hydrogel particles can be an alternative method for minimally invasive drug delivery. When compared to their macroscopic analogues, microgels and nanogels have some advantages. Their small size enables them to be needle-injectable, and they provide a large surface area for bioconjugation. This allows facile natural clearance and improved penetration through tissue barriers [117].

Recently, microgels and nanogels formed by the self-assembly of short peptides have emerged as promising biomaterials and have exhibited enormous potential in many biomedical fields, including controlled drug release. An important example was reported by Liu and Xing et al., who described stimulus-responsive short peptide nanogels for controlled intracellular drug release [118]. They presented an environmentally responsive nanogel system, which self-assembles with DOX and P-glycoprotein inhibitor. This nanogel exhibits acid-sensitive properties for controlled drug release, while simultaneously inhibiting the efflux function of P-glycoprotein. Thus, this system effectively reverses multi-drug resistance of cancer drugs and results in improved tumor treatment [118].

## 5. Bioadhesive Properties and Biofunctionalization of Self-Assembling Peptide Hydrogels

In the previous sections, we presented the peptide-self-assembly process, drug release triggers, and the stability and mechanical properties of peptide-based supramolecular hydrogels, illustrated with various examples. The functionalization of peptide hydrogels offers new tools to both fine-tune the mechanical properties and to tailor the biomimetic properties. In this section, the functional tailoring of peptide-based hydrogels is explored, showcasing the strategies recently adopted for improving the effectiveness and enhancing the application of these hydrogels in drug delivery.

### 5.1. Peptide-Based Hydrogel Adhesion to Surfaces for Local Drug Delivery

Just like the overall size of the hydrogel, the bioadhesive properties are also an important factor when selecting the gel delivery route. As mentioned above, there are several biological barriers, such as the intestinal epithelium and mucosa, that limit the adhesion properties of the hydrogels. Over the years, extensive efforts have been made to develop bioadhesive hydrogels for local drug delivery.

The possibility of using self-assembling peptide-based viscous solutions and hydrogels as mucoadhesives for improved delivery of drugs to mucosal surfaces, such as the buccal, nasal or ocular areas, was investigated by Saiani et al. [119]. Based on the previous work reported by Zhang et al. [83], the group developed a range of self-assembling β-sheet-forming peptides, eight amino acids long, with alternating hydrophobic and hydrophilic amino acids. This design allowed the creation of hydrogels with tailored properties. Exploring the properties of these hydrogels, they investigated the possibility of using these biomaterials as mucoadhesives for local drug delivery. Focusing on the Phe–Glu–Phe–Glu–Phe–Lys–Phe–Lys octapeptide, they studied the release of two commercial drugs, lidocaine, which was soluble under the conditions used in this work, and flurbiprofen, which was insoluble [119]. The results showed that the addition of lidocaine resulted in stiffening of the samples. Drug retention was not generally favored, as both the drug and the peptide carry charges of the same sign. Nonetheless, improved drug retention was observed in the cases of the stiffer samples. The addition of flurbiprofen to the samples did not change the mechanical properties of the gels. For the samples with weak mechanical properties, the presence of the drug reduced their resistance to physical erosion by salt solution flow. Conversely, when the initial mechanical properties of the hydrogels were sufficiently high, the addition of flurbiprofen significant increased their resistance to erosion. The toughness of the gel is a key factor for its ability to maintain its structure and avoid fracture during use and after adhesion. Considering the respective area in which the hydrogel is to be applied, the peptide can be functionalized in order to improve the mucoadhesive properties. For example, considering the negative charges of the ocular mucosa, the rational design of cationic peptide-based gels is promising for prolonging the ocular residence time, as it provides a better mucoadhesive property via the electrostatic interaction with negatively charged mucin. Liu et al. recently used cationic peptide as the molecular hydrogelator for generating supramolecular hydrogels for extended ocular drug delivery [120]. Furthermore, Taka et al. reported on the ocular co-delivery of timolol and brimonidine from a self-assembling peptide hydrogel for the treatment of glaucoma [121]. They evaluated the in situ gel forming self-assembling peptide ac–(RADA)_4_–CONH_2_ as a carrier for both pharmaceuticals, with the goal of creating an alternative to conventional eye-drops.

Another strategy used to improve bioadhesion is the incorporation of a catechol moiety (i.e., DOPA) into the hydrogel structure. Catechol chemistry is a convenient approach for preparing functionalized hydrogels with good adhesive properties, as this motif can bind to various inorganic surfaces through various non-covalent interactions, such as coordination, hydrogen bonding and hydrophobic effects. They can also bind to organic surfaces through the reaction of an oxidized version, the metabolite DOPA-quinone, with either primary amines or thiols [122,123,124]. Recently, Xue, Li and Cao et al. reported on smart adhesive peptide nanofibers for cell capture and release [125]. The group developed a smart cell capture and release system based on adhesive self-assembling peptide nanofibers, by integrating a DOPA motif into the peptide. The peptide can self-assemble into nanofibers at physiological pH to establish strong binding to both cells and the substrate surface for cell capture. Slightly raising the pH triggers disassembly of the peptide fibers, and the isolated peptides with a single DOPA group can adhere to either cells or the substrate, but not both. This process leads to the detachment of cells. The group showed that this technique is efficient and can be widely used.

### 5.2. Biomimetic Functionalization

Extracellular matrices (ECMs) are three-dimensional (3D) networks of macromolecules that are particularly rich in peptides and carbohydrates, which display several bioactive sites available for cellular binding, internalization, and regulating their homeostasis [126]. Functionalized hydrogels that mimic natural ECMs can serve as 3D tissue mimetic scaffolds to support cell adhesion, proliferation and differentiation [127]. Self-assembling peptides (SAPs) are promising supramolecular hydrogelators for both cell culture and biomedical applications, and by extension, drug delivery [128,129]. SAPs possess the distinctive advantage of sharing the chemical composition of commonly employed functional motifs, thus allowing their functionalization using similar synthetic strategies. These motifs can be obtained from the vast literature, through protein docking analysis in silico, through screening of synthesized peptide libraries, and by screening phage display against a specific target (e.g., cell, drug) [130,131]. RGD from fibronectin and IKAV from laminin are among the most used functional motifs in SAP applications, owing to their short length, simple synthesis and ubiquitous distribution in the ECM of living tissues [132,133,134].

In a previous work, Tsutsumi et al. developed a self-assembling peptide named E1Y9 (Ac–Glu–Tyr–Glu–Tyr–Lys–Tyr–Glu–Tyr–Lys–Tyr–NH_2_) that responds to calcium ions to form supramolecular nanofibers, and then eventually hydrogels [127]. In a follow-up to this work, the nanofibers of E1Y9 were non-covalently functionalized with fiber-binding peptides (called p1, p2 and p3) identified from a screen of a phage peptide library [135]. RGDS-conjugated forms of the peptides p1, p2 and p3 significantly enhanced the adhesion of 3T3-L1 cells to E1Y9 nanofibers. The proliferation of 3T3-L1 cells was greatly improved through this noncovalent modification of E1Y9. This work successfully demonstrated that peptides that can selectively bind to the supramolecular nanostructures of SAPs, and that such structures can be obtained from a diverse peptide library. The isolated peptides can be applied to the functionalization of SAP materials by conjugation with functional molecules such as bioactive sequences [135]. Non-covalent functionalization methods can make it easier to add several functional groups to supramolecular hydrogels by changing only the material-binding peptides with functional units [136].

There are several biomedical applications for these kinds of functionalized peptide-based hydrogels, and there has been an increased research effort to maximize their potential in the last few years. Yang, Wang and Zhu et al. recently reported self-assembling peptide hydrogels functionalized with the LN-derived peptide epitope IKAV and the BDNF (brain-derived neurotrophic factor)-mimetic peptide epitope RGI, and their potential enhancement in peripheral nerve regeneration [136]. The group found that the dual-functionalized SAP hydrogels promoted adhesion to rat Schwann cells, myelination and neurotrophin secretion in vitro, and successfully bridged a 10 nm gap in an in vivo model of a sciatic nerve defect in rats [136]. Ghosh et al. reported a neuroprotective injectable sulfo-functionalized peptide hydrogel able to mimic the ECM, with a view to repairing brain injury [137]. The group showed that their hydrogel is capable of repairing brain injury by mimicking an ECM-like environment and providing neuroprotection to the damaged neurons [137]. Koksch et al. developed peptide hydrogels to mimic the ECM by functionalizing coiled-coil peptides with cellular binding sequences or carbohydrate ligands (mannose), and by utilizing the multivalency and compatibility of coiled-coil assemblies [138].

## 6. Drug Release Profiles

As we are focusing on the application of supramolecular peptide hydrogels in drug delivery, the “drug release” concept must be clarified: it consists of the migration of pharmacological solutes from the hydrogel matrix to the system surface and then to the surroundings [139]. The physical–chemical and structural characteristics of the hydrogel fibril network, loaded drugs and release medium determine the drug delivery profile. In fact, we can take advantage of multiple stimuli to control these properties and thus control drug delivery, as discussed earlier [140,141]. In this section, we will address the primary aspects and kinetics involved in drug release from hydrogels, since the respective drug delivery behaviour is still a topic of discussion [141].

### 6.1. Introduction to Drug Release Kinetics of Peptide Hydrogel Matrices

In swelling polymers, the molecular drug release mechanism is a combination of drug diffusion and polymeric network relaxation. Modelling the solute movement in these materials is thus more complicated. In 1987, Ritger and Peppas suggested that Equation (1), previously proposed by themselves to relate the amount of drug released to time [142], can also be applied to swelling systems (not exceeding 25% expansion) for the first 60% of release data [143]:

(1) $$\frac{M_{t}}{M_{\infty }}=kt^{n}\;$$

where $M_{t}$ is the mass of drug released at time t, $M_{\infty }$ is the mass of drug released as time approaches infinity, k is a constant involving the properties of the drug and macromolecular network system and n is the diffusional exponent which translates the transport mechanism [143,144]. The diffusional exponent depends on the type of transport, hydrogel geometry and peptide polydispersity. When it takes the value of 0.5, the drug is released by Fickian diffusion (case-I transport). Alternatively, when the value equals 1, erosion and/or swelling of the material dominates the release process (case II-transport). When n is between 0.5 and 1, there is more than one mechanism at work in the system—non-Fickian drug release [25,142,143]. We are now able to compare drug release characteristics of different hydrogel matrices. As such, it is important to determine the value of k, which indicates the initial kinetics of drug release (over a unit of time), and the half-life of release ($t_{1/2}$), which gives information about the time it takes for $\frac{M_{t}}{M_{\infty }}$ to reach 50% [25]. Our own research group recently reported some examples of hydrogels where the kinetics of drug release were modelled using this equation. One of these examples was a bolamphiphilic tetrapeptide, and the other was a naproxen-capped dipeptide folate receptor ligand composed of non-natural amino acids. In these cases, non-Fickian diffusion of the encapsulated drug from the hydrogel network was observed [145,146].

**Figure 7:**
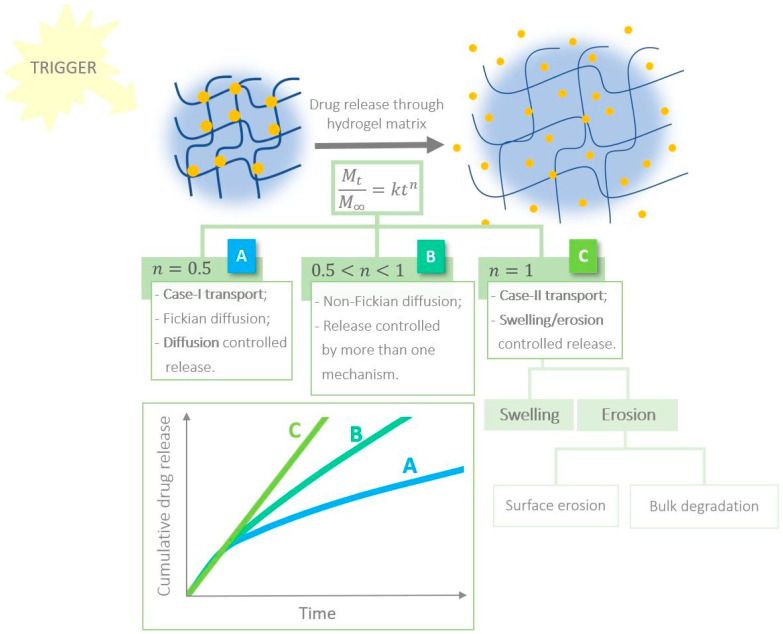
Schematic representation of the different drug release kinetics from a hydrogel matrix and the aspects to consider in case-II transport.

Many peptide hydrogels mentioned within this review are stimuli responsive. However, they all share a common feature of degradability, by virtue of the natural origin of the building blocks [17]. This term refers to the process by which the fibrils begin to release their content to the outside, in the presence of appropriate external thermodynamic conditions [139]. When this process is the predominant drug release mechanism, it is important to distinguish between the two types of degradation: surface erosion vs. bulk decomposition [147,148]. For example, in Section 2, we saw that MMP enzymes can participate in the decomposition of MMP-substrate-motif-incorporating hydrogels. When the rate of the original enzyme–substrate reaction is faster than the rate of enzyme transport, we are dealing with surface erosion; otherwise, bulk degradation is operating [147]. These concepts are outlined in Figure 7. In fact, the in-depth study of target cellular parameters (e.g., MMP concentration) in predicting the degree of degradability of these materials is central to controlling solute release. The reader should note that there are numerous aspects that define the drug release profile of biodegradable stimuli-responsive materials, since the local conditions are constantly changing. The coexistence of diffusion, chemical reactions, moving boundaries, changes in volume, biological interactions and dissolution of amino acids make the characterization of the drug delivery process highly complex [140]. There are excellent reviews addressing this issue in detail [139,140,147], so we will next highlight some recent advances in this area of peptide hydrogels for drug delivery.

In order to precisely regulate the release rate of PTX, Chakroun designed several PTX–peptide conjugates with different critical aggregation concentrations (CAC) [149]. CAC translates the predisposition of the hydrogel filaments to dissociate. Thus, it is understood that the compound with the highest CAC exhibits the fastest release rate, while the compound with the lowest CAC has shown the slowest conjugate release. Taking this into account, a two-phase mechanism for pro-drug release has been inferred. The release of PTX–peptide conjugate began at the hydrogel–medium interface, where filament dissociation and monomer diffusion occurred due to the entry of water (swelling) and osmotic pressure [147], respectively. Because of gel shrinkage, the second phase occurred through bulk dissociation to achieve thermodynamic equilibrium with the surroundings [149]. In this case, a small surface area accounted for the slow linear release profile of the drug-peptide conjugate. Despite the conventional preference for sustained drug release, burst release may also be desirable, depending on the drug administration strategy [150]. As such, some methods have been developed to modulate the sol–gel state of the matrix. For example, it is possible to define the lifetime of pH-responsive peptide hydrogels by programming the pH profile of the surrounding medium. This is accomplished using biocatalytic activity to produce acid/base without the introduction of external triggers: the higher the enzyme concentration, the faster the state transition of peptide nanofibers [150,151]. In an approach aimed at tackling prostate cancer, He et al. designed a gelling pro-drug in the form of peptide-BTL conjugate (indomethacin–GFFK(bicatulamide)EH) [21]. The release behaviour of the conjugate was studied at pH 7.4, 6.5 and 5.5. Burst release was not observed at any pH, but it was found that the release rate increased with decreasing pH. The authors argue that this behaviour was due to the protonation of histidine residues at lower pH. This phenomenon would have resulted in fibril disruption owing to the non-coordination of the monomers with zinc ions. However, it was demonstrated that the release of the conjugate was not only due to the relaxation of the gel network, but also to the diffusion of the pro-drug, since the calculated diffusion coefficient n was in the range of between 0.5 and 1 [21,27]. In similar investigations, a slow and prolonged drug delivery is desired [33]. It is possible to reduce the rate of content release through several adjustments [25], such as in vitro drug extraction for short periods before in vivo application, heterogeneous distribution of the loaded drug, hydrogel surface modification, drug-free coating application and changes in peptide nanofibre morphology/composition [25,150]. In fact, studies that report the application of self-assembling peptides in drug-loaded polymer encapsulation to achieve a degradation lag are already published [152]. Furthermore, if we want to obtain a $t_{1/2}$ greater than 1 day we can change the morphology and composition of the nanofibers, by introducing elements that increase the electrostatic and hydrophobic interactions (increasing $t_{1/2}$ to 2–3 days) or by inserting cleavable covalent bonds (increasing $t_{1/2}$ to ≈1 week) between the drug and the fibres [25]. Nonetheless, Choe and Yun chose not to conjugate the drug with the self-assembling peptide. Instead, they loaded the Fmoc-FF-based hydrogel with an anti-inflammatory drug, indomethacin (IDM) [153]. The value of n increased a the decrease in the IDM content in the hydrogel, with super case II-transport when the IDM concentration was 0.2 wt.%, case II-transport when the IDM concentration was 0.4 wt.% and non-Fickian diffusion when the IDM concentration was 0.5 wt.%. Therefore, for low concentrations of IDM, erosion predominates, which was proved when investigating the stability of the Fmoc-FF based hydrogel in phosphate buffer. Interestingly, at an advanced stage, drug release rate was reduced for all IDM concentrations, and it was concluded that Fickian diffusion mechanism was operating (n < 0.45) [25]. Hence, a system was developed with a biphasic release profile, tunable through changes in the loaded drug concentration [153].

### 6.2. DOX as a Model Drug: Different Hydrogel Matrices for Anticancer Drug Loading and Release

Despite the array of existing treatments, cancer is one of the leading causes of death worldwide, being responsible for about 10 million deaths in 2018, according to the WHO [33,154]. In cancer nanomedicine, supramolecular peptide hydrogels stand out due to their numerous advantages, such as biocompatibility, tuneable mechanical properties, and stimuli-responsive controlled drug delivery. The latter point can circumvent the problem of systemic toxicity of most chemotherapeutic drugs conventionally used, such as doxorubicin (DOX) [155,156,157,158]. This drug, in particular, causes irreversible damage to the myocardium, and therefore it is desirable to incorporate it into biomaterials, in order to increase its accumulation in tumour tissues, while reducing the associated side effects, without compromising its therapeutic efficiency [159,160,161,162]. As a drug used in the treatment of a wide range of cancers, DOX is often adopted as a model drug in investigations of nanosystems for drug delivery [159]. Since review articles on this topic lack information about the impact of the matrix on the performance of a nanosystem in drug delivery, here we propose to make a comparison and critical evaluation of the most recent studies on peptide hydrogels for DOX loading and release.

In a novel approach, Karavasili’s team developed the ac–(RADA)_4_–CONH_2_ hydrogelator (Table 1, structure A) in order to load DOX and curcumin in different compartments, taking advantage of their distinct physical–chemical characteristics [163]. Alternatively, Mei developed two similar peptides, IDM-1 (indomethacin–GFFYGRGDH) [164] and 1-RGDH (Nap–GFFYGRGDH) [163] (Table 1, structures B and C, respectively), to load DOX and then later release it in an acidic environment [164,165]. The diffusion exponent n of the DOX release profile was evaluated for each experiment, as well as the kinetic constant k, for both Mei research works, and are summarized in Table 1. The DOX release profile from the ac–(RADA)_4_–CONH_2_-based hydrogel does not depend on pH, and at physiological conditions the release is dominated by DOX diffusion, since an extremely low n value was calculated. AFM images showed that DOX resided at the periphery of the nanofibers, not interacting with them to a large degree, which supports the previous results. Accordingly, 80% of DOX was released in the first 4 h, with a plateau occurring after 1 day [163]. On the contrary, when the IDM-1 peptide as used as the matrix, there was a pH-responsive DOX release: under acidic conditions, the drug diffusion effect increased, compared to erosion/swelling influence, and the initial release k of DOX was much faster. The greater DOX diffusion at low pH can be then attributed to the disruption of electrostatic interactions between DOX and the carboxylic acid groups of IDM-1, which are protonated at acidic pH [165]. Regarding the 1-RGDH self-assembling peptide, two release mechanisms were observed: non-Fickian diffusion in the first 24 h and (super) case-I transport thereafter. Furthermore, the DOX initial release rate k increased under higher proton strengths and this phenomenon can be explained in the same way as for the IDM-1 molecule [165]. Over the period of 24–168 h, DOX release was completely controlled by diffusion (super case-I transport) due to the presence of naphthalene. It is well-stablished that this *N*-capping group is a gelation inducer [166], and therefore it can be presumed that 1-RGDH-based hydrogel resisted degradation, particularly as a large amount of solute was released during the first day.

For all the tested acidities (pH 5.5, 6.5 and 7.4), the initial DOX release rate k of 1-RGDH was greater than that of the peptide IDM-1. These observations were expected, since it is known that the self-assembly propensity conferred by the naphthalene moiety can be a hindrance to the interactions between the nanofibres and DOX, decreasing its binding capacity [164,166]. At pH 7.4, case-I transport was observed for the ac–(RADA)_4_–CONH_2_ peptide, and non-Fickian diffusion for the IDM-1 and 1-RGDH peptides was observed over the first 24 h. This indicates that gel erosion exerted an influence on DOX release in the case of the IDM-1 and 1-RGDH matrices, contrary to ac–(RADA)_4_–CONH_2_ nanofibres. This can be explained by the electrostatic repulsion of negatively charged aspartic acid and histidine residues at physiologic pH [165]; fibril dissociation for both IDM-1 and 1-RGDH-based hydrogels resulted in an increase in the diffusional coefficient n to values between 0.5 and 1. This effect was not observed with the ac–(RADA)_4_–CONH_2_ system, where only diffusion acted, due to the drug location being close to the surroundings. At pH 5.5, 1-RGDH peptide showed a significantly higher n value than that of the IDM-1 peptide over the first 24 h. After a faster initial DOX release from the 1-RGDH hydrogel, a larger water intake occurred, relative to the IDM-1 hydrogel; thus, swelling will have led to mesh enlargement, promoting further DOX release [167].

Overall, it can be concluded that all the peptides studied show great potential for controlled DOX delivery, depending on the desired release profile. The corresponding hydrogels satisfy the fundamental rules for controlled DOX delivery: firstly, drug stabilization in the gel matrix, either through covalent conjugation, physical entrapment, or co-assembly with the hydrogel-forming peptides; secondly, DOX-controlled release (by tuneable diffusion or swelling/erosion processes) triggered by a stimulus that is specifically present in the target tissue; and finally, enhanced DOX potency [164,165,168,169]. However, one should note that a large proportion of the drug release results from its diffusion, which is a fast process. In the future, the electrostatic interactions used in these systems may be supported by cleavable covalent bonds in order to reduce diffusion and protect the drug [167,170]. Changes in the *C*-terminus residue can also be carried out since it has recently been reported that it plays a determining role in the rate and extent of the peptide-fibre interactions [171]. In fact, continuous studies on the kinetics, dynamics and biophysical properties of these and other nanosystems are critical in their translation into clinical practice.

## 7. Conclusions and Future Direction

In this review, we have summarized the drug delivery applications of supramolecular peptide hydrogels, with particular emphasis on the latest developments in the field. Drug delivery systems have been assessed in terms of their self-assembly processes, drug release profiles, mechanical and bioadhesive properties, while strategies for accelerating the rate of drug release ‘on demand’ have also been considered. We have also cast a spotlight on the mechanisms and kinetics of drug release.

We have seen how peptides present significant advantages over alternative drug delivery systems, owing to an inherent non-toxicity, biocompatibility, and ease of synthesis and functionalization. Local administration of therapeutic agents can overcome problems associated with low solubility and adsorption of drug molecules. Toxicity and side-effects can be reduced (relative to systemic delivery) as reduced doses can be used. We have seen how a local drug depot can be designed to respond to its surroundings in stimuli-responsive hydrogels, alongside more conventional diffuse-controlled release.

Peptide hydrogels still suffer from a number of limitations when compared with materials derived from carbohydrates or polymeric materials [172,173]. They generally have low tensile strength, which can be a problem for some applications. In addition, there are often loading issues with drugs that are particularly hydrophobic. These limitations are offset to a certain by the specific benefits of using peptides discussed within this review. Hybrid materials, able to combine the most favorable properties of different classes of material (including chemically cross-linked materials), may well be attractive targets in future research. Furthermore, the progress required to improve the properties of peptide hydrogels may lie in the leverage of artificial intelligence (AI) and computational techniques involving molecular modelling, molecular dynamics (MD) simulations and machine learning technologies—such techniques are already playing a key role in the design of supramolecular materials. Overall, the future goals in this research area should be development of systems where the therapeutic release can be sustained over longer periods (where required), or further tailored to the specific requirements of the patient. It is expected that some of these drug delivery systems will be progressed towards clinical applications in the near future.

## Figures and Tables

**Table 1:**
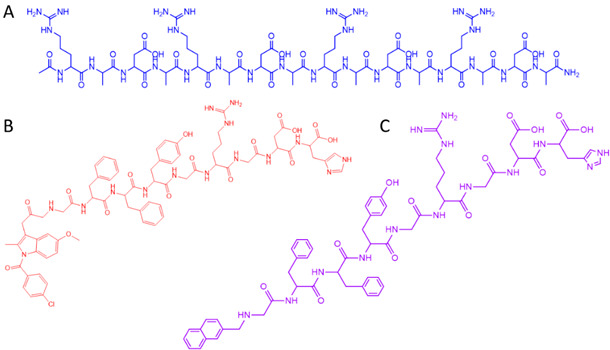
Kinetic constants and diffusion exponents for ac–(RADA)4–CONH_2_, IDM-1 and 1-RGDH, according to the pH conditions tested in the corresponding experiments. (Data from [163,164,165]).

			k	n	Drug Release Mechanism	Reference
**ac–(*RADA*)_4_–*CONH*_2_**	pH 7.4	0–≈450 h	–	0.141	Case-I transport	[165]
***IDM*-1**	pH 5.5	0–24 h	0.0219	0.6282	Non-Fickian difusivo	[166]
	pH 6.5		0.0068	0.7975	Non-Fickian diffusion	
	pH 7.4		0.0046	0.7857	Non-Fickian diffusion	
**1-*RGDH***	pH 5.5	0–24 h	0.0342	0.8084	Non-Fickian diffusion	[167]
		24–168 h	0.1374	0.2265	Case-I transport	
	pH 6.5	0–24 h	0.0185	0.7946	Non-Fickian diffusion	
		24–168 h	0.0766	0.2039	Case-I transport	
	pH 7.4	0–24 h	0.0127	0.7768	Non-Fickian diffusion	
		24–168 h	0.0471	0.2189	Case-I transport	

## Abbreviations

3D: three-dimensional; AFM, atomic force microscopy; AI, artificial intelligence; BDNF, brain-derivative neurotrophic factor; Bhcmoc, 6-bromo-7-hydroxycoumarin-4-ylmethoxycarbonyl; CAC, critical aggregation concentration; Cbz, benzyloxy carbonyl; CD, circular dichroism; CGC, critical gelation concentration; CNB, carboxynitrobenzyl; CNTs, carbon nanotubes; CPPs, cell penetrating peptides; DOX, doxorubicin; ECM, extracellular matrix; EM, emodin; FITC, fluorescein isothiocyanate; Fmoc, fluoronylmethoxycarbonyl; FTIR, Fourier-transformed infrared spectroscopy; G’, storage modulus; GEM, gemcitabine; GSH, glutathione; HCPT, 10-hydroxycamptothecin; IDM, indomethacin; L-DOPA, 3,4-dehydroxy-L-phenylalanine; LCST, low critical solution temperature; LF, bovine lactoferrin; MD, molecular dynamics; MMP2, metalloproteinase 2; MMP7, metalloproteinase 7; NAP, naphthoxyacetic acid; NDIP, napthalenediimide; Nvoc, nitroveratryloxycarbonyl; Pas, peptide amphiphiles; PEG, polyethylene glycol, PNIPAM, poly(*N*-isopropylacrylamide); PTX, paclitaxel; PXRD, powder x-ray diffraction; SAPs, self-assembling peptides; SATO, S-aroylthiooxime; SC(*o*NB), *o*-nitrobenzyl-protected phosphonated serine residue; SPIONS, super-paramagnetic iron oxide nanoparticles.
